# Affective Out-World Experience via Virtual Reality for Older Adults Living with Mild Cognitive Impairments or Mild Dementia

**DOI:** 10.3390/ijerph20042919

**Published:** 2023-02-07

**Authors:** Maria Matsangidou, Theodoros Solomou, Fotos Frangoudes, Konstantinos Ioannou, Panagiotis Theofanous, Ersi Papayianni, Constantinos S. Pattichis

**Affiliations:** 1 CYENS Center of Excellence, Nicosia 1016, Cyprus; 2 Department of Computer Science, School of Natural & Applied Sciences, University of Cyprus, Nicosia 1678, Cyprus; 3 Archangelos Michael Elderly People Nursing Home/Rehabilitation Centre for Patients with Alzheimer (AMEN), Nicosia 1022, Cyprus

**Keywords:** virtual reality, older adults, mild cognitive impairment, dementia, human-centered technologies, user experience, affective computing, emotional experiences

## Abstract

Older adults with cognitive impairments may face barriers to accessing experiences beyond their physical premises. Previous research has suggested that missing out on emotional experiences may affect mental health and impact cognitive abilities. In recent years, there has been growing research interest in designing non-pharmacological interventions to improve the health-related quality of life of older adults. With virtual reality offering endless opportunities for health support, we must consider how virtual reality can be sensitively designed to provide comfortable, enriching out-world experiences to older adults to enhance their emotional regulation. Thirty older adults living with mild cognitive impairment or mild dementia participated in the study. Affect and emotional behavior were measured. The usability and the sense of presence were also assessed. Finally, we assessed the virtual reality experiences based on physiological responses and eye-tracking data. The results indicated that virtual reality can positively enhance the mental health of this population by eliciting a positive affective state and enhancing their emotional regulation. Overall, this paper raises awareness of the role of virtual reality in emotion elicitation, regulation, and expression and enhances our understanding of the use of virtual reality by older adults living with mild cognitive impairments or mild dementia.

## 1. Introduction

Dementia is an umbrella term for progressive symptoms which are linked to some form of cognitive decline and can emerge as a reaction to co-existing symptoms of emotional distress [1]. Most commonly, dementia is diagnosed when the severity of cognitive impairment affects the social and/or occupational functioning of the older adult. There is, however, an intermediate state between normal cognition and dementia known as mild cognitive impairment, during which the person shows objective evidence of a reduction in cognitive abilities (i.e., learning, memory, language fluency, visuospatial ability, executive functioning, and psychomotor ability), but maintains functional abilities [2]. Mild cognitive impairment is diagnosed when only one type of cognitive ability is impaired, while when more than one of the abilities is impaired, a dementia diagnosis is provided [3].

The traditional conceptualization of dementia attempts to treat the disease with prescription therapies, to manage the symptoms and maximize the cognitive capabilities of the person living with dementia [4]. However, the World Health Organization set a priority to reduce the risk of cognitive decline by providing non-pharmacological interventions, to help delay, prevent, or treat related diseases [5,6,7,8]. This is also reflected in recent approaches, that recognize the clinical manifestation of dementia as a blend of neurological deficits and psychosocial factors and explore alternative means for interventions. It has been suggested that the interventions for individuals living with mild cognitive impairment or dementia should emphasize a person-centered philosophy of care [1,9], and implement personalized formulation-led interventions [10,11,12,13]. For example, autobiographical life story reviews, with an emphasis on emotional memories, can meet emotional needs and manage the behavioral and psychological symptoms of dementia (BPSD) [14]. This is because emotions were found to influence a wide range of cognitive processes and have been linked to increased well-being [15,16,17].

Over the last two decades, virtual reality (VR) has slowly begun to have a presence in the healthcare sector introducing innovative non-pharmacological ways of delivering treatment and care for people with mild cognitive impairment or dementia [18,19,20,21,22]. Particularly, it has been used with people living with dementia as an intervention tool to reduce anxiety and/or agitated behaviors [23,24] and elicit positive emotions [25,26]. For example, people living with dementia exposed to a virtual nature experience using a semi-immersive VR system [23] showed a reduction in anxiety and agitation after the VR sessions. More recently, in a similar work [24] which followed a single-case design and used a fully immersive VR system, it was shown that VR can be an effective intervention to decrease agitation in people living with dementia. The evidence from both of the above studies, even though limited, supports the efficacy of VR for such purposes, and therefore the technology is promising and warrants further exploration.

Brimelow et al., in a pair of studies [26,27], showed that using 360-degree videos of travel destinations, natural environments, etc., in VR with people with mild cognitive impairment or mild dementia can reduce feelings of apathy and depression. In a similar approach, which was coupled with an interactive virtual botanical garden [28], it was also shown that VR can improve mood and calm people with cognitive impairments living in residential aged care. The above studies only focused on content related to travel destinations or outdoor environments, and not so much on other more intimate events and locations encountered in daily life, such as urban locations or traditional or religious-related settings that can potentially be more reminiscent for people with mild cognitive impairment or with dementia. Similar results were reported in a study wherein a randomized control trial was compared to a conventional approach (i.e., watching a movie), and VR resulted in a more significant reduction in stress in the participants at the end of the sessions [22]. The results, however, were based on a relatively small sample (14 participants) and the results could be skewed by some external factors as well, such as the medications used by a number of the participants.

The use of VR with people with mild cognitive impairment was also found to be effective as a pain distractor, and also made participants happier and more relaxed and elicited reminiscences [29]. The presented content in the above study comprised immersive video clips of popular locations in Canada. This can potentially limit the creativity and ability of participants to associate the experienced virtual environments (VE) with other locations they might have encountered in their past. This can be avoided with the use of computer-generated content which also allows the enhancement of the VEs with more personalized elements. VR with the use of computer-generated content has also shown greater effects in improving mood compared to other more traditional mediums, such as large-screen TVs [30].

We believe that VR is effective with this population as it can offer multisensory interactions and feedback (e.g., visual, auditory, tactile) to the user. Current research suggests that VR can be a reliable, feasible, and acceptable solution, which can promote engagement and provide an enjoyable experience for people with mild cognitive impairment or mild dementia. In addition, there is a need to craft personalized experiences that take into consideration the preferences and skill level of each user [25,31,32,33]. As such, computer-generated content can be used along with VR to provide such experiences and simulate a variety of locations across different themes to meet the needs of older adults with cognitive impairments.

Building on the above findings, and to examine if VR can play a fundamental role in eliciting emotional experiences in older adults with mild cognitive impairment or mild dementia, we examine the following hypothesis:

**H1.**  *VR will elicit positive emotions in people with mild cognitive impairment or mild dementia.*

**H2.**  *VR will reduce the negative emotions of people with mild cognitive impairment or mild dementia.*

## 2. Materials and Methods

### 2.1. Participants

Thirty (males = 11, 36.6% and females = 19, 63.3%) people with mild cognitive impairment or mild dementia and age range between 55 and 87 and mean age of 69.03 years (SD = 10.66) participated in the study. Participants had no prior experience using VR. Of those, 3 (male = 1, 33.3%, females = 2, 66.6%) with a mean age of 73.33 (SD = 11.93) and mild cognitive impairment refused to wear the headset due to the material factors. All participants had a normal or corrected vision and no history of severe motion sickness. Additional exclusion criteria were the diagnosis of moderate or severe dementia or another major neurological or psychiatric disorder. Ethical approval was sought from the national health bioethics research committee. The diagnosis was confirmed using the Mini-Mental State Examination (MMSE) [34]. A commonly shared threshold for mild cognitive impairment is set at 26/27 points and <24 for mild dementia [34,35,36,37,38], our participants were diagnosed with mild cognitive impairment or mild dementia, and they had a mean MMSE score of 25.23 (SD = 2.16).

### 2.2. Study Design and Procedure

**Figure 1:**
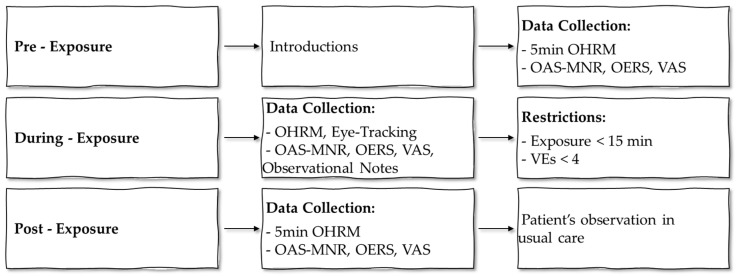
Study design and procedure.

**Figure 2:**
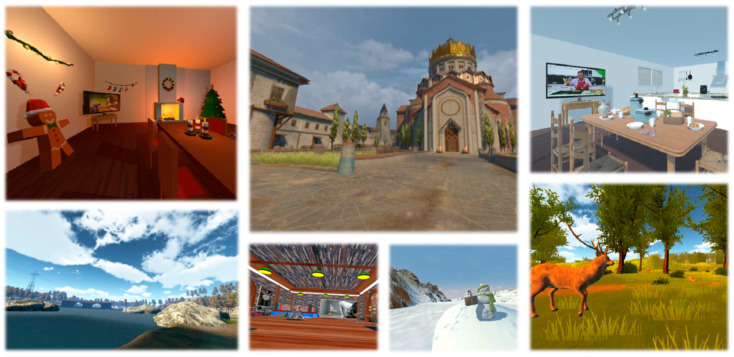
Snapshots of representative VEs.

The study design emerged from a systematic review that examined the feasibility of VR for people with neurological disorders and dementia [1,39], as well as discussions with experts in the field of geriatric care and eHealth. The VEs that were used in the developed system were selected using a multi-step process. All aspects of the design process are documented and published [40]. Data were collected within a month and included quantitative-subjective responses and physiological metrics. The study design and procedure can be seen schematically in Figure 1. As a first step, we recorded “pre-exposure” quantitative measures before the VR session. Then, the participants were provided with an A3-sized sheet of paper, as a “Menu” with pictures of the available VEs to choose from (see Figure 2 which presents an example of VEs from each category. For the complete menu of VEs please refer to [40]). Each participant could choose up to three VEs to be exposed to. This allowed participants to experience different environments and examine the effects each environment can have on their mood and overall cognitive state.

Afterward, VR was introduced to the participants. To prevent adverse effects, such as dizziness, associated with VR, a maximum total duration of 15 min was suggested for experiencing all VEs. This corresponds to roughly 5 min per environment, which allows enough time to explore each one, and prevents boredom in the case of a single protracted experience. During the VR session, various “during-exposure” quantitative measures were taken to assess the emotional state of participants during the experience. As part of the post-exposure measures, mild cognitive impairment or mild dementia patients filled in quantitative data related to their experience, followed by a short interview. On average, each session lasted 45 min.

### 2.3. Materials

- Eye-tracking data. Participants’ eye-related metrics were tracked using an eye tracker embedded in the VR headset at 10 Hz. These data allowed the analysis of the behaviour and gaze patterns of participants, thus providing the opportunity to gain a better understanding of what they were experiencing.
- Observed Emotion Rating Scale (OERS) [41]. The scale offers direct observation of the time (1 = never, 2 = less than 16 s, 3 = 16–59 s, 4 = 1–2 min, and 5 = more than 2 min) spent expressing five affect types: pleasure; anger; anxiety; sadness; and general alertness, before, during (i.e., after experiencing each of the VEs), and after the session. An HCI researcher marked for how long a participant expressed each of the five affect types during each section of a session. For example, participants could have shown anger for less than 16 s, felt sadness for 16–59 s, and had general alertness for more than 2 min. The scale was selected to evaluate how the emotions of participants changed during the intervention.
- Visual Analogue Scale (VAS) [42]. VAS was used to record the self-assessed emotional state of participants during the session. The participants were asked to point to the emoji (0 = happy and 5 = sad) that matched their emotional state before and after the session. During the session the participants were asked to refer to the emotion they were feeling, the researcher read out loud each emotion (0 = happy and 5 = sad).
- Slater-Usoh-Steed Questionnaire (SUS) [43]. The scale assesses the level of presence and immersion using seven questions on a 7-point Likert scale (e.g., 1 = being somewhere else and 7 = being in the VE) and was recorded after the session. The questionnaire can provide insights into the experience of participants during the session and the feasibility of the technology to “transfer” participants to different environments.
- System Usability Scale (SUS) [44]. The scale evaluates the system’s usability using ten questions rated on a 5-point Likert scale (1 = strongly disagree to 5 = strongly agree). The scale was administered after the use of the system to assess the overall usability of the system.
- Semi-structured interviews. Semi-structured interviews were conducted by two researchers. We examined the “technology acceptance” of VR using a combination of questions related to usability, practicality, and immersiveness of the system. For example, participants were asked whether they felt immersed in the experience, whether they forgot about the physical boundaries of the room, as well as whether the VR headset was comfortable to wear. Questions were also asked regarding the emotional effects of VR and based on observations made during the session.

### 2.4. Apparatus

The VR system was developed using the Unity3D game engine and the 3D models were retrieved from the Unity Asset Store and repurposed to run on a VIVE Pro Eye VR system. The VR content was streamed on a laptop screen, mirroring the participants’ real-time view. Gaze was tracked through the HMD’s eye tracker and visualized in real-time using a ray that was only visible on the laptop screen, and thus not distractive for the participant.

## 3. Results

### 3.1. Presence, Immersion, and Systems’ Usability

High rates of presence and immersion (max score of 7) were reported by all participants (M = 5.87, SD = 0.90). High rates of system usability, with an average score of 77.40 (SD = 11.78), were also reported.

### 3.2. Eye-Tracking

Descriptive statistics indicated that most people with mild cognitive impairment or mild dementia were interested in environments relevant to travel, for example visiting Venice (n = 11) and Nauplio Island (n = 8). This was followed by familiar places (n = 8), such as a living rooms. Six participants requested to experience religious places or sceneries (n = 6). It is worth noting that some of the participants reacted as if the VE was a real holy place. Specifically, 4/6 of the participants crossed themselves while entering the temple environment (a common practice when entering a church). This supports the high level of immersion and presence. Open fields such as forests and orchards were also highly chosen (n = 7), while parks were selected by another five participants.

In addition to the above, various eye-related metrics were measured (i.e., gaze origin and direction, pupil diameter and position). Based on these metrics we were able to identify the objects participants were looking at (e.g., trees, lakes, birds, etc.), the number of times they fixated on each object, the time that passed before they looked at an object for the first time, the number of times the subject looked at the object, etc. These objects were then grouped into thematic categories (e.g., water surfaces, flora, appliances, etc.). Based on these categories, aggregated data were computed of the participants’ interaction with objects within each category and averaged across the number of VEs these objects were placed in.

**Figure 3:**
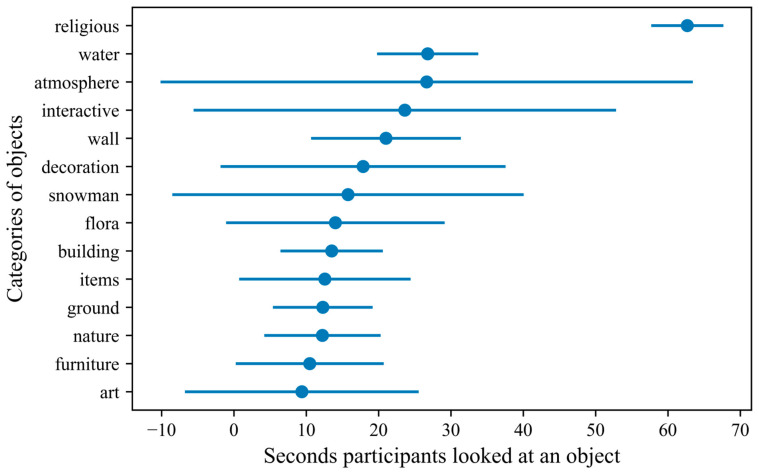
The thematic groups of objects participants fixated on, based on the average duration for each within the VEs (the error bars indicate standard deviation).

The category of objects participants fixated on the most on average within a scene was religious objects (Figure 3). This was primarily due to the temple within the VE with the same name, and its oversized and imposing presence within that VE. Following this were objects related to water surfaces (e.g., lakes, rivers, fountains, etc.), the atmosphere (e.g., sky, clouds, etc.), and interactive elements (e.g., content on TV, hot-air balloons moving in the sky, etc.). Most of the participants (M = 82%, SD = 27%) looked at the same objects across the VEs they experienced.

A Shapiro–Wilk test on pupil diameter, after the removal of outliers, indicated a normal distribution (*p* = 0.47). Therefore, a Student’s t-test was performed on the pupil’s diameter values before and after exposure to each VE. The results showed a significant difference between the two with an increase in the diameter of the pupil during exposure (t(47) = −5.337, *p* =< 0.0001). The effect was greater in VEs related to patient-familiar content.

### 3.3. Affective Experiences in VR

A range of sources of data were analyzed to identify the affective experiences in VR by people with mild cognitive impairment or mild dementia. As can be seen from the results detailed below, VR usage was associated with many positive emotions.

#### 3.3.1. Observed Emotion Rating Scale (OERS)

Friedman test indicated that ratings of pleasure significantly differed between before, during, and after VR exposure, χ^2^(2) = 25.391, *p* = 0.000. Wilcoxon signed-rank tests revealed a significant increase in pleasure from before to during VR exposure Z = −3.792, *p* = 0.000 and from before to after VR exposure Z = −3.612, *p* = 0.000. There was no significant difference between during and after VR exposure.

Ratings of anger significantly differed between before, during, and after VR exposure, χ^2^(2) = 12.000, *p* = 0.002. Wilcoxon signed-rank tests revealed a significant decrease in anger between before and after VR exposure Z = −2.214, *p* = 0.027 and from during to after VR exposure Z = −2.585, *p* = 0.010. There was no significant difference between before and during VR exposure.

Ratings of anxiety/fear significantly differed between before, during, and after VR exposure, χ^2^(2) = 27.815, *p* = 0.000. Wilcoxon signed-rank tests revealed a significant decrease in anxiety/fear from before to during VR exposure Z = −3.482, *p* = 0.000 and from before to after VR exposure Z = −3.559, *p* = 0.000, and between during and after VR exposure Z = −1.933, *p* = 0.053.

Similarly, the ratings of sadness were significantly different between before, during, and after VR exposure, χ^2^(2) = 23.247, *p* = 0.000. Wilcoxon signed-rank tests revealed a significant decrease in sadness from before to during VR exposure Z = −3.020, *p* = 0.003 and a significant decrease in sadness from during to after VR exposure Z = −4.532, *p* = 0.000. There was no significant difference between before and after VR exposure.

Finally, ratings of general alertness significantly differed between before, during, and after VR exposure, χ^2^(2) = 30.545, *p* = 0.000. Wilcoxon signed-rank tests revealed a significant increase in general alertness from before to after VR exposure Z = −2.324, *p* = 0.020, from before to after VR exposure Z = −4.679, *p* = 0.000, and during to after VR exposure Z = −2.077, *p* = 0.038. There was no significant difference between before and during VR exposure.

**Figure 4:**
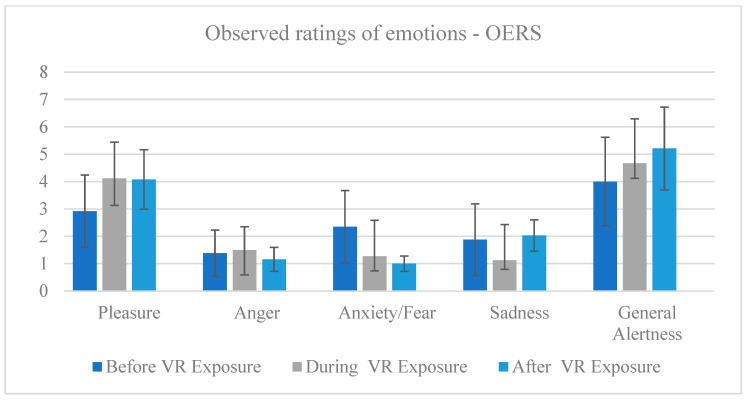
Observed ratings of emotions before, during, and after VR exposure using the OERS.

There was also no significant difference in ratings of anger before, during, and after VR exposure (Table 1, Figure 4).

#### 3.3.2. Visual Analogue Scale (VAS)

**Figure 5:**
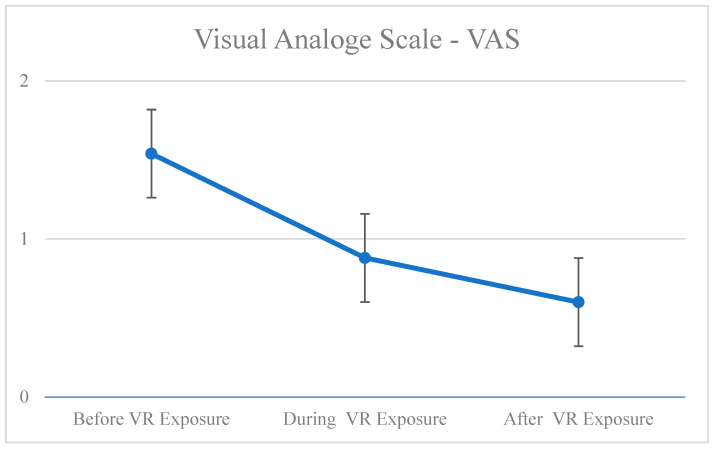
Emotions before, during, and after VR exposure using VAS.

Friedman test indicated that there was a significant reduction in the negative emotions between before, during, and after VR exposure, χ^2^(2) = 22.160, *p* = 0.000. Wilcoxon signed-rank tests revealed a significant decrease in negative emotions (and a significant increase in positive emotions) from before to during VR exposure Z = −3.253, *p* = 0.001 and from before to after VR exposure Z = −3.841, *p* = 0.000. However, there was no significant difference between the emotional state of the person during and after the VR exposure (Table 2, Figure 5).

### 3.4. Interviews

Thematic analysis was used to analyze the transcribed interview [45]. Data were analyzed at the semantic level, focusing on explicit meanings. An essentialist epistemology guided the analysis and interpretation of the data, which assumes a largely unidirectional relationship between meaning and experience [45]. The overall aim of the analysis was to provide a detailed account of a group of themes within the data, based on our specific research questions. To address the research questions, three researchers critically discussed and reviewed the data to discover patterns, which were then coded and refined into themes.

Based on the thematic analysis of the interviews, two core themes and six subthemes emerged. People with mild cognitive impairment or mild dementia discussed “Virtual Reality’s Impact on Health” and “Virtual Reality and Material Properties”. Table 3 schematically presents the core and subthemes of the thematic analysis.

#### 3.4.1. Virtual Reality’s Impact on Health

Three subthemes were discovered in relation to the immersive virtual reality experiences: memories evoke feelings, natural reactions, and interactions, and exposure reduces anxiety.

Memories evoke feelings. Following exposure to the VR experience, most people with mild cognitive impairment or mild dementia (21/27) reminisced positively about memories of past events, relatives and family gatherings, geographical origins, and travels. In most cases, people with mild cognitive impairment or mild dementia reported that the VEs resembled familiar places and events and reminded them of their earlier days. Even though the system was not completely personalized, we managed to match the interests and memories of the participants through the careful selection of the VE by involving representative users in the selection and evaluation processes (hidden for blind review). This is not the first time that a non-personalized VE has been perceived as familiar and evoked memories for people living with dementia [24,46]. As a result, we conclude that high ratings of presence and similar elements to a memory can help older adults with mild cognitive impairment or mild dementia recall their past experiences.

Extract 1:

“The room was decorated for Christmas. There was a fire in the fireplace. Tom and Jerry Christmas episodes were playing on the TV. I was reminded of the good old days. When I was a mother and my husband was still around. Back when our kids were young. It made me feel melancholic, but in a good way” (People with mild cognitive impairment 7).

Natural reactions and interactions. In line with the presence questionnaire outcomes and according to our observations, the majority of people with mild cognitive impairment or mild dementia (25/27) behaved naturally during the experiences, as if they were physically present. In one instance, a person with mild dementia tried to grab a flower, while others tried to touch the sand under their feet, and another tried to grab the snow and make a snowman. A repetitive action was observed in a virtual church environment. There was a strong impression that people with mild dementia or mild cognitive impairment reacted as if they were in a real holy place when they visited the VE. Particularly, and as mentioned earlier, four out of six participants crossed themselves as they entered the temple.

Extract 2:

“A lot of snow fell there. Although I tried to make a snowman, I could not touch the snow. How come? It snowed, but it wasn’t cold…” (People with mild dementia 12).

Exposure reduces anxiety. People with mild cognitive impairment or mild dementia (18/27) reported reduced anxiety after experiencing VR. The scale outcomes in “Affective Experiences in VR” provide further support for this observation. In particular, it was found that people with mild cognitive impairment or mild dementia were experiencing some sort of daily anxiety, but once immersed into the VR system they managed to relax. This is not the first study proving the effectiveness of VR to reduce anxiety in people with dementia. In general, research has suggested that VR can be an effective solution for the reduction of behavioral and psychological symptoms of dementia [47,48].

Extract 3:

“It seemed like something was wrong with me today. I was feeling gloomy. It felt like a huge stone was sitting on my chest. As soon as you arrived, I thought to myself, “There is no way I would be a good candidate”. But then you took me to the lake, and I feel calm, and the stone has been lifted from my chest…” (People with mild cognitive impairment 19).

#### 3.4.2. Virtual Reality and Material Properties

Concerning VR’s material properties, three subthemes were identified: acceptability, practicality, and social presence.

Acceptability. The majority of the participants (27/30) found the system comfortable to wear.

Extract 4:

“The device felt comfortable on my head, like wearing some sort of bulky glasses. More like wearing some kind of bionic eyes through which you can see a whole new world. [A researcher asked whether it was claustrophobic or stressful]. No, I do not think it felt that way at all… In fact, it was the opposite. I felt relaxed rather than stressed, and instead of feeling claustrophobic, the feeling was more of a freedom.” (People with mild dementia 5).

However, and as fully immersive VR systems surround the user’s view completely, three people with mild cognitive impairment (but not with mild dementia) refused to wear the headset (3/30). Participants reported that the system evoked feelings of anxiety and was claustrophobic. In the past, there has been evidence of similar responses in people living with dementia [49]. It has been reported that handheld VR systems (i.e., holding the HMD with your hands) are preferred by patients with mild dementia [25].

Extract 5:

“Oh, my dear! [Her hands snag the VR headset, and she pulls it over in agony] Oh my, I’m so sorry, but I can’t! I’m sorry, but I cannot wear this… Please understand that I cannot wear this… it’s so stressful and claustrophobic… I can’t do it, sorry, I can’t.” (People with mild cognitive impairment 6).

Practicality. Even though most of the participants enjoyed VR, two reported that the headset was heavy to wear, and the visuals appeared blurry (2/27). The participants were both petite-figured women with mild dementia. Based on our observations, we were able to determine that the headset did not fit well. According to a previous study, an exoskeleton might reduce uncomfortable feelings and make the VR headset more practical [49]. Additionally, this solution might be able to reduce the incidence of catastrophic behavior mentioned above.

Extract 6:

“Well, I liked it, but it was too heavy and big for me. I had difficulty breathing because it wasn’t staying in place and fell into my face blocking my nose. […] The colors seemed to fade away as it fell into my face, so it would be great if you made it to feet so I could see all the colors clearly. Having to push it in and hold it in the right spot tired me” (People with mild dementia 3).

Social Presence. In contrast to the commonly held belief that VR is an isolating experience [50], we discovered that VR can be a space where social presence can exist. This means that VR can create a sense of being with another person by utilizing digital interfaces and human–computer interaction principles. Particularly, exposing people with mild cognitive impairment or mild dementia to virtual spaces where avatars exist and behave like humans can enhance their sense of belongingness and social presence. Specifically, our participants (15/27) commented very positively on the avatars that walked or behaved around them in the virtual worlds. In a previous study, people with dementia experience loneliness, which predicts their condition worsening [51], so using avatars in VEs may enhance the sense of being with others, thereby reducing loneliness.

Extract 7:

“It was nice to be in there. I was surrounded by many people. It was a pleasure to have them with me at the church… to see how many people are coming to the church. And then we visited Venice. I enjoyed seeing the gondolas and the gondoliers around the city and people passing shops and restaurants… It is not time for the launch yet, so the restaurants were empty…” (People with mild dementia 30).

## 4. Discussion

In this proof-of-principle study, we examined the use of VR technology to enrich the emotional health of older adults with mild cognitive impairment or mild dementia. We particularly examined how VR can be used to evoke, regulate, and express emotions in older adults with mild cognitive impairment or mild dementia. Our findings were in line with our hypotheses H1 and H2, proposing that VR can enhance the emotional well-being of people living with mild cognitive impairment or mild dementia.

In detail, H1 is accepted since the results demonstrated a significant increase in pleasure and happiness. Our participants’ emotions were positively enhanced during the VR exposure. The fact that these positive emotions persisted after the exposure is worthy of special attention. Our findings are consistent with the general bibliography which suggests that VR can have a positive impact on older adults with cognitive impairments [24,25,52].

VR is often used as a tool to elicit emotional responses, and it has been shown that such responses can be reflected in pupil dilation. For example, in Chen et. al., [53] participants experienced five different scenes in VR, each with the purpose of triggering a different emotion (i.e., happiness, fear, anxiety, sadness, disgust). As a result, both positive and negative emotions caused dilation in their pupils. Our results also support this effect, as the participants’ pupils dilated during exposure to the VEs, verifying the positive effects recorded through the questionnaires.

Similarly, H2 is accepted since there was a significant decrease in participants’ anxiety, fear, and sadness. This is in line with previous research which suggested that spending time in outdoor environments can reduce agitation, pacing, and exit-seeking behaviours, as well as the use of psychotropic medications for people living with dementia [54,55,56,57]. Additionally, our results suggested that VR was able to increase the general alertness of the person and so reduce apathy and lack of motivation which is commonly found as a symptom in older adults with mild cognitive impairment or mild dementia [26,58].

It is worth noting that at the beginning of the VR intervention, anxiety and fear may be evoked because of the material factors of the system and the unfamiliarity the people with mild cognitive impairment or mild dementia have with VR. However, within half a minute in the VEs, their anxiety was alleviated.

In general, our study revealed the positive effects VR can have on people with mild cognitive impairment or mild dementia, including improved mental health through the elicitation of positive affective states and emotional regulation. There were, however, some negative side effects as well. In particular, some of our participants reported feeling a high degree of presence which resulted in a side effect of sadness and/or disorientation when transitioning from the virtual to the real world. This side effect has been observed in previous studies which documented that some people living with dementia might feel disoriented after VR exposure [49,59]. To reduce the negative side effects, during our interventions, the therapist that was present guided participants back to the real world by discussing their experience.

## 5. Limitations, Conclusions, and Future Work

Our study examined and showed the short-term effects VR can have during interventions with people with mild cognitive impairment or mild dementia. However, a large-scale, longitudinal study is needed to identify whether VR has potential long-term effects. A second limitation is the small sample size. We hope to explore these opportunities for a broader population in the future, as well as the impacts they can have on individualized care and evaluating affective responses among people with moderate dementia and severe dementia. Even so, this study provides insight into the different aspects of interaction and hardware design. Furthermore, it gives insight into the challenges that such equipment encounters in the deployment phase, based on the reported accessibility and practicality issues. Overall, this study contributes to the growing body of research on using digital technology for people living with mild cognitive impairment or mild dementia.

A key motivation for the current study is the potential use of VR to improve the health-related quality of life and emotional health of older adults living with mild cognitive impairment or mild dementia. Through the observations among participants and their behavior during the sessions, we have shown that similar emotions and reactions can be evoked every time a VE is visited. Therefore, VR has the ability to provide participants with similar experiences within a VE they enjoy on-demand and across different sessions. This ability is also not affected by extraneous variables, such as weather, etc., which could prevent similar experiences in the real world. The use of computer-generated content also allows the creation of more personalized content that caters to the needs of different people. This can be further enhanced with the inclusion of people’s personal items (e.g., photographs) in the VEs. However, and based on the findings of a recent study by Berrett et al., it is crucial to understand that older adults living with mild cognitive impairment or mild dementia differ on their functional and emotional levels and so the systems must be sensitively designed to meet individual requirements, so as to offer a tailored solution to each patient [60].

Nevertheless, some negative side effects were reported once the VR session was over (e.g., sadness and disorientation). As mentioned above, to reduce the negative side effects, during our interventions the therapist that was present guided participants back to the real world by discussing their experience. In the future, other technologies, such as the passthrough feature of modern VR and mixed-reality headsets, can be used to ease the transition of participants from one world to another.

Lastly, the use of wearable devices and sensors, such as eye-tracking devices, motion sensors, etc., provides an opportunity to track the emotional responses of participants in real-time. Based on such measurements, in the future the content can be further adapted, even providing real-time customizations for the delivery of personalized experiences, tailored to each individual and based on their actions during a VR-based experience.

## Figures and Tables

**Table 1:** Observed ratings of emotions before, during, and after VR exposure using OERS.

Affect	*p*		M	Mdn	SD	Phase	*p*
Pleasure	0.000	Before	2.92	3.00	1.32	Before–during	0.000
		During	4.12	4.00	0.99	Before–after	0.000
		After	4.08	4.50	1.09	During–after	0.869
Anger	0.002	Before	1.38	1.00	0.85	Before–during	0.257
		During	1.50	1.00	0.91	Before–after	0.027
		After	1.16	1.00	0.44	During–after	0.010
Anxiety/fear	0.000	Before	2.35	1.50	1.32	Before–during	0.000
		During	1.27	1.00	0.53	Before–after	0.000
		After	1.00	1.00	0.28	During–after	0.053
Sadness	0.000	Before	1.88	1.00	1.31	Before–during	0.003
		During	1.12	1.00	0.33	Before–after	0.160
		After	2.03	2.00	0.57	During–after	0.000
Alertness	0.000	Before	4.00	5.00	1.62	Before–during	0.020
		During	4.67	5.00	0.55	Before–after	0.000
		After	5.21	6.00	1.51	During–after	0.038

**Table 2:** Emotions before, during, and after VR exposure using VAS.

	*p*		M	Mdn	SD	Phase	*p*
Emotional state	0.000	Before	1.54	2.00	0.71	Before–during	0.001
		During	0.88	1.00	0.66	Before–after	0.000
		After	0.60	0.75	0.63	During–after	0.096

**Table 3:** People with mild cognitive impairment and dementia themes.

Virtual Reality’s Impact on Health	Virtual Reality and Material Properties
Memories evoke feelings	Acceptability
Natural reactions and interactions	Practicality
Exposure reduces anxiety	Social Presence

## Data Availability

Not applicable.
